# Juvenile Cataract and Chronic Diarrhea: A Single Etiology

**DOI:** 10.7759/cureus.79760

**Published:** 2025-02-27

**Authors:** Maria I Risto, Patrícia Sobrosa, Maria Vilela, Luciana Sousa

**Affiliations:** 1 Internal Medicine, Hospital de Santa Luzia - Unidade Local de Saúde do Alto Minho, Viana do Castelo, PRT

**Keywords:** cerebrotendinous xanthomatosis, chenodeoxycholic acid, cyp27a1 mutations, diarrhea, juvenile cataracts

## Abstract

Cerebrotendinous xanthomatosis (CTX) is a rare, autosomal recessive metabolic disorder characterized by an abnormal bile acid synthesis pathway, resulting in cholesterol accumulation that can deposit in different tissues, causing a variety of clinical manifestations. We present the clinical case of a young male patient referred to Internal Medicine for investigation of chronic diarrhea, later diagnosed with bilateral cataracts - classic symptoms of CTX, which led to his diagnosis. The authors aim to emphasize the importance of considering a single etiology in young patients with various seemingly unrelated signs/symptoms.

## Introduction

Cerebrotendinous xanthomatosis (CTX) is a rare autosomal recessive disorder caused by a mutation in the cytochrome P450 family 27 subfamily A member 1 (CYP27A1) gene, leading to abnormal accumulation of cholesterol and its derivatives in tissues, particularly in the central nervous system, eyes, tendons, and blood vessels.

In North America, the estimated prevalence ranges from one in 72,000 to one in 150,000 [1], whereas in Europe, it is between one in 134,970 and one in 461,358 [2]. These values are likely underestimated due to delays in diagnosis. In Portugal, no prevalence data is currently available.

Clinical manifestations include gastrointestinal symptoms in childhood, such as diarrhea and neonatal jaundice, early-onset juvenile cataracts, xanthomas in adolescence, and progressive neurological deterioration in adulthood [3,4]. Cataracts are the initial symptom in 75% of affected individuals, usually presenting in the first decade of life [5].

Diagnosis of CTX is established through a combination of clinical evaluation, biochemical findings, and genetic confirmation of a CYP27A1 gene mutation [5]. Biochemical markers indicative of CTX include elevated serum cholestanol levels, typically exceeding 10 mg/L, with normal or low cholesterol levels. Additionally, bile alcohols are significantly increased in blood, bile, and urine [6]. While genetic confirmation of CYP27A1 mutations provides a definitive diagnosis, these biochemical findings serve as strong supportive evidence, especially since the clinical manifestations of CTX can mimic those of other disorders.

Treatment aims to prevent symptoms in asymptomatic individuals and halt progression in those already affected, thus avoiding complications. It involves chenodeoxycholic acid administration to normalize cholesterol levels and alleviate neurological symptoms, with effectiveness monitored through serum cholestanol levels [6]. For cataracts, surgical intervention is required.

Differential diagnosis should exclude monogenic disorders such as congenital diarrhea, sitosterolemia, and familial hypercholesterolemia, depending on the clinical presentation. Due to their rarity, these diagnoses are also challenging to consider.

This condition has a poor prognosis as it is often diagnosed late when neurological damage has already occurred. This case describes the clinical presentation of a young patient with CTX, a condition that is both difficult to diagnose and frequently delayed, underscoring the importance of clinical suspicion.

## Case presentation

We present the case of a 21-year-old male patient with a family history of first-cousin parents and a previous diagnosis of ureteropelvic junction obstruction (UPJO), which was evaluated in Urology. He was referred to Internal Medicine by his primary care physician (PCP) due to several years of chronic diarrhea.

The diarrhea reportedly began after an episode of acute gastroenteritis at approximately three years of age, characterized by three daily loose, brown stools, generally following meals and occasionally accompanied by abdominal pain. The patient denied nocturnal stools, blood or mucus in the stool, visible parasites, or systemic symptoms.

Key findings from the workup included anxiety episodes that worsened diarrhea, a diet rich in saturated fats and salt with no intake of fruits or vegetables, mild iron deficiency without other hematological abnormalities, normal lipid and liver profiles, normal sedimentation rate, and normal C-reactive protein levels. Antibodies for celiac disease (anti-tissue transglutaminase IgA and anti-endomysial IgA) were negative (Table 1).

**Table 1:** Analytical values

Parameter	Result	Reference value
Hemoglobin	16.4 g/dL	13.2-17.2 g/dL
ESR (Erythrocyte sedimentation rate)	2 mm	2-8 mm
CRP (C-reactive protein)	0.4 mg/dL	<0.5 mg/dL
Total cholesterol	145 mg/dL	<200 mg/dL
c-HDL (High density lipoprotein)	51 mg/dL	>60 mg/dL
Ferritin	393.8 ng/mL	21.81-274.66 ng/mL
Bile acids	8.60 μmol/L	>8.10 μmol/L
AST (Aspartate aminotransferase)	19 U/L	10-32 U/L
AST (Aspartate aminotransferase)	15 U/L	10-33 U/L
GGT (Gamma-glutamyltransferase)	20 U/L	6-42 U/L
ALP (Alkaline phosphatase)	64 U/L	35-105 U/L

Initially, the clinical presentation was explained by diet and irritable bowel syndrome. The patient was referred to Nutrition and placed on loperamide without improvement. Upon preclinical assessment, the patient was diagnosed with bilateral cataracts. The cataracts were posterior subcapsular in type, a feature that is diagnostic of CTX, and large enough to require surgical intervention. The patient underwent phacoemulsification, the standard technique of cataract removal, followed by intraocular lens implantation.

Considering the unusual etiology and possibility of having one unifying cause for juvenile cataracts and chronic diarrhea, CTX was considered. The patient was evaluated in the hospital's Metabolic Disorders and Genetics clinics, and clinical, biochemical, and molecular diagnosis of CTX was made with the identification of a homozygous variant in the CYP27A1 gene. The identified variant was c.1024C>T (p.Arg342Trp), at 2: 141,657,974 (GRCh37). The mutation results in an amino acid change from arginine to tryptophan at position 342 of the protein. Treatment with chenodeoxycholic acid 250 mg three times daily was initiated, and the patient remains under follow-up. There was an improvement in the biochemical values following the treatment, demonstrating a positive response to the therapy.

## Discussion

CTX was first documented in 1937 [7]. It is a rare and underdiagnosed condition with an insidious onset and variable symptoms, often leading to diagnostic delays. Bilateral ocular involvement, diarrhea, and atherosclerosis are among its manifestations, as well as neurological impairment and tendon xanthomas, the latter two being rare before age 20 [8]. Systemic symptoms typically present earlier than neurological ones [6].

The chronic diarrhea observed in this patient is explained by bile acid malabsorption, while juvenile cataracts are a classic early sign of the disease [8]. Diagnosis is based on clinical presentation, biochemical findings, imaging studies, and genetic testing for confirmation [6].

MRI is the modality of preference for brain evaluation in CTX and is helpful in disease follow-up [6]. Classic imaging findings of CTX include periventricular white matter hyperintensities, cerebral peduncle, globus pallidus, and cerebral and cerebellar atrophy. These alterations are typically imaged with fluid-attenuated inversion recovery (FLAIR) MRI sequences, which are very sensitive to white matter alterations and are crucial for the identification of early manifestations of the disease. In addition, hyperintensities in the dentate nuclei and adjacent white matter, visible on FLAIR images, are always present and are considered markers of CTX [8]. While FLAIR imaging is especially helpful in revealing these findings, T1-weighted and T2-weighted imaging are also helpful in tracking the disease's progression and providing complementary information regarding structural brain changes.

Genetic testing for CYP27A1 mutations is crucial for confirming the diagnosis of CTX. Initial therapy with chenodeoxycholic acid achieves normalization of the level of cholestanol to avoid disease in asymptomatic individuals and prevents disease progression in affected individuals [6]. Additionally, it alleviates gastrointestinal and neurological symptoms without reversing established neurologic impairment [1,6,9]. In 2009, a synthetic form of chenodeoxycholic acid, known as chenodal, was reapproved in the United States by the Food and Drug Administration (FDA) for the treatment of gallstones. Chenodal has also been utilized as a treatment for CTX and was approved by the FDA for the treatment of CTX as an orphan drug in the United States. Studies show that patients who began treatment at or above the age of 25 had worse outcomes, with significantly more limited mobility and greater cognitive impairment than patients who began treatment under the age of 25 [6]. Another bile acid, cholic acid, has been utilized in the treatment of young children with CTX. While chenodeoxycholic acid remains the drug of first choice, cholic acid is employed in specific circumstances since it lacks the risk of hepatotoxicity, a possibility with chenodeoxycholic acid in some situations. Treatment, therefore, is essential for a favorable prognosis [8].

The patient’s family history of consanguinity supports the hypothesis of a hereditary disease, likely with a recessive inheritance pattern such as CTX [1].

## Conclusions

The case demonstrates the importance of searching for rare disorders such as CTX in children presenting with an uncommon constellation of symptoms, i.e., protracted diarrhea and infantile cataracts. The diagnosis was based on clinical presentation, having a homozygous mutation of CYP27A1 and supported by a family history of consanguinity. Early diagnosis and treatment of the condition prevent serious complications, particularly prior to the onset of neurological deterioration. Genetic counseling is also significant among family members and individuals affected since it offers a service for extended family screening to identify asymptomatic carriers or genetically affected persons who may be put under early prophylactic therapy. Prevention of recurrence can also be dealt with through the application of reproductive strategies such as prenatal diagnosis and preimplantation genetic testing to make the therapeutic intervention early and improve long-term outcomes.
